# Conserved and novel responses to cytokinin treatments during flower and fruit development in *Brassica napus* and *Arabidopsis thaliana*

**DOI:** 10.1038/s41598-018-25017-3

**Published:** 2018-05-01

**Authors:** Victor M. Zuñiga-Mayo, Cesar R. Baños-Bayardo, David Díaz-Ramírez, Nayelli Marsch-Martínez, Stefan de Folter

**Affiliations:** 1 Unidad de Genómica Avanzada (LANGEBIO), Centro de Investigación y de Estudios Avanzados del Instituto Politécnico Nacional (CINVESTAV-IPN), Irapuato, 36824 Guanajuato, Mexico; 2 Departamento de Biotecnología y Bioquímica, CINVESTAV-IPN, Irapuato, 36824 Guanajuato, Mexico

## Abstract

Hormones are an important component in the regulatory networks guiding plant development. Cytokinins are involved in different physiological and developmental processes in plants. In the model plant *Arabidopsis thaliana*, cytokinin application during gynoecium development produces conspicuous phenotypes. On the other hand, *Brassica napus*, also known as canola, is a crop plant belonging to the Brassicaceae family, as *A. thaliana*. This makes *B. napus* a good candidate to study whether the cytokinin responses observed in *A. thaliana* are conserved in the same plant family. Here, we observed that cytokinin treatment in *B. napus* affects different traits of flower and fruit development. It increases ovule and seed number, affects stamen filament elongation and anther maturation, and causes a conspicuous overgrowth of tissue in petals and gynoecia. Furthermore, cytokinin recovers replum development in both wild type *B. napus* and in the *A. thaliana rpl ntt* double mutant, in which no replum is visible. These results indicate both conserved and novel responses to cytokinin in *B. napus*. Moreover, in this species, some cytokinin-induced phenotypes are inherited to the next, untreated generation, suggesting that cytokinins may trigger epigenetic modifications.

## Introduction

*Brassica napus*, also known as oilseed rape or canola, is a crop plant belonging to the Brassicaceae family*. B. napus* is widely cultivated around the world due to the edible oil that is extracted from its seeds.

**Figure 1:**
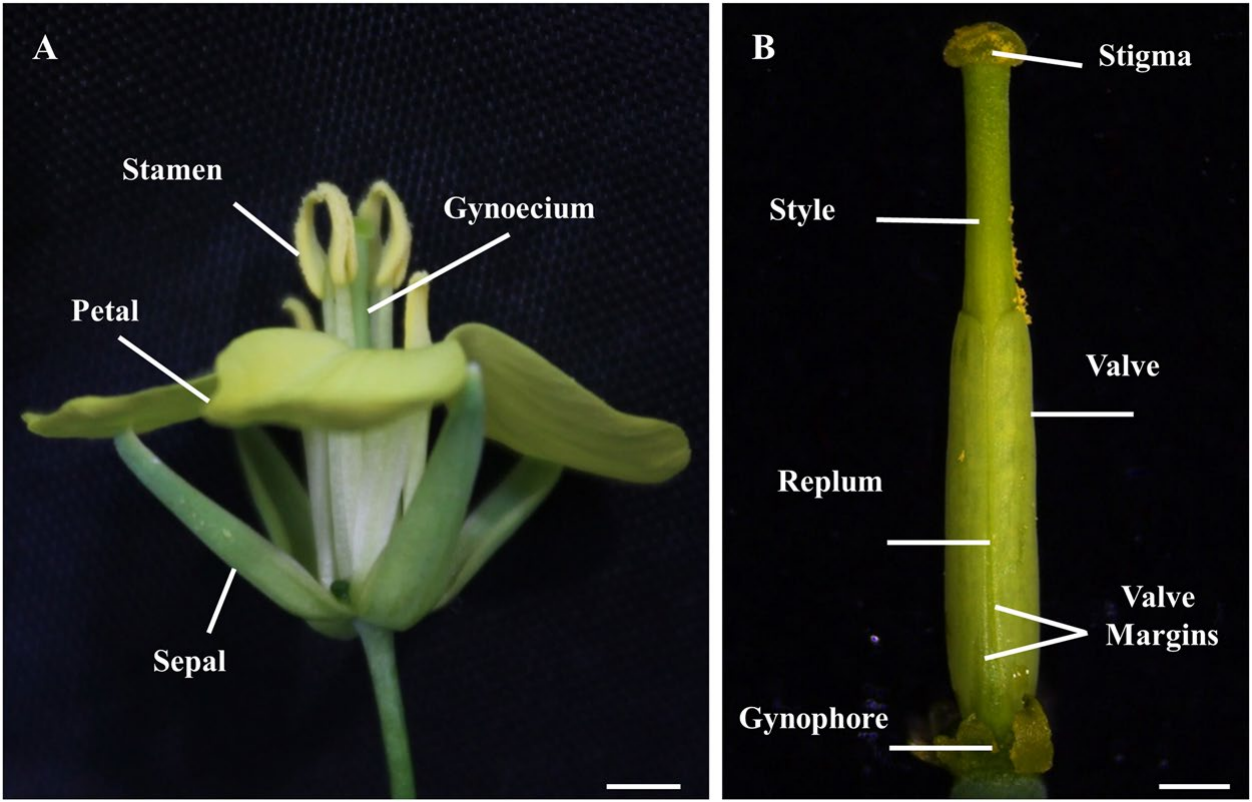
Overview of a *Brassica napus* flower and gynoecium. (**A**) *Brassica napus* (Canola) flower at anthesis stage. (**B**) *Brassica napus* gynoecium at anthesis stage. Scale bars: (**A**) 2 mm; (**B**) 1 mm.

The flower of *B. napus* (Fig. 1A) is very similar to a flower of *Arabidopsis thaliana*, which is also a member of the Brassicaceae family. Both flowers have four sepals, four petals, six stamens (four long and two short stamens), and a pistil in the center consisting of two carpels. In both species, the pistil (or gynoecium) can be divided into different developmental axes. The apical-basal axis consists out of four domains, from the apical to the basal region: the stigma formed by a single layer of specialized cells, the style (a solid tissue), followed by the ovary containing the ovules, transmitting tract and the septum, and finally the gynophore, which is a short stalk-like structure connecting the pistil with the rest of the plant (Fig. 1B). After fertilization, different parts of the pistil grow and differentiate to give rise to the fruit, where the medio-lateral axis can be divided into three domains: the valves, which are the ovary walls protecting the developing seeds, the replum, which separates the valves and is connected to the septum to divide the fruit into two halves, and finally the valve margins located between the valves and the replum where the fruit will open in order to disperse the seeds^1–3^.

Hormones have an important impact on plant development throughout the life cycle plants. One of the most studied plant hormones, besides auxins, are cytokinins. Cytokinins regulate different developmental and physiological processes in plants such as shoot meristem formation and maintenance, root development, organ formation, response to both abiotic and biotic stress, seed germination, delay of senescence, and fruit and seed development, among others^4–8^.

The role of cytokinins in fruit and seed development has been studied for several decades. In the 80’s, several studies were carried out in soybean, mung bean and oilseed rape where it was observed that cytokinin application prevented flower abortion or increased flower number resulting in improved yield^9–13^. Afterwards, manipulation of endogenous cytokinin levels was studied. The first approach was to increase endogenous cytokinin levels by ectopic expression of *ISOPENTENYL TRANSFERASE* (*IPT*) genes that catalyze the rate-limiting step in cytokinin biosynthesis. For this, several promoters have been used, however, although some positive effects have been achieved, such transgenic plants also had both morphological and physiological negative effects^14–18^. The other approach followed was to reduce cytokinin degradation through down-regulation of *CYTOKININ OXIDASE/DEHYDROGENASE* (*CKX*) genes. In this regard, natural variation has been identified where some *CKX* genes are down-regulated or an RNAi method has been used to silence these genes^7,19,20^.

Recently, in *A. thaliana* it has been reported that cytokinins, in addition to the number of ovules per pistil, also regulate other aspects of pistil and fruit development such as medial tissue proliferation, apical-basal patterning at early stages of the developing pistil, and valve margin differentiation at more mature stages. Cytokinins also affect stamen development reducing the filament length and pollen grain production^21–26^.

As described above, most studies on cytokinins and fruit development are focused on improving crop yields. However, few studies have evaluated the role of cytokinins in other aspects of fruit development. In this work, we analyzed the response to cytokinin treatments in *B. napus* plants, different traits of flower and fruit development were evaluated, besides the number of ovules and seeds. We found conserved and novel responses to cytokinin treatments during flower and fruit development compared to *A. thaliana*. Furthermore, the results suggest that in order to achieve a fruit with more seeds it is not enough to make a pistil with more ovules, at least not in *B. napus*. Also, cytokinin appears to be regulating replum development in both *B. napus* and *A. thaliana*. Moreover, we observed that some phenotypes originally caused by exogenous cytokinin application in *B. napus* were inherited to the offspring, suggesting that somehow cytokinins are able to induce changes at the epigenetic level.

## Results

### *Brassica napus* presents similar responses to exogenous cytokinin applications as *Arabidopsis thaliana*

Exogenous cytokinin application causes, in the *A. thaliana* gynoecium, tissue proliferation that arises from the replum with stigmatic papillae at the tip. In the stamens, it produces a reduction in filament length and pollen grain production^22^. Furthermore, in both *A. thaliana* and rice, *ckx* mutants have been identified that produce more ovules and seeds^19,21^. *B. napus* has several industrial applications. Therefore, we decided to determine whether exogenous cytokinin application in this species has similar effects as those observed in *A. thaliana*. For this, *B. napus* plants were treated with the cytokinin BAP (6-Benzylaminopurine), using two different types of application. On the one hand, inflorescences were treated five days a week for three weeks, with a 200 µM BAP solution, and another set of plants was treated with a single application of lanolin paste with 500 µM BAP on the inflorescences and analyzed four weeks after.

**Figure 2:**
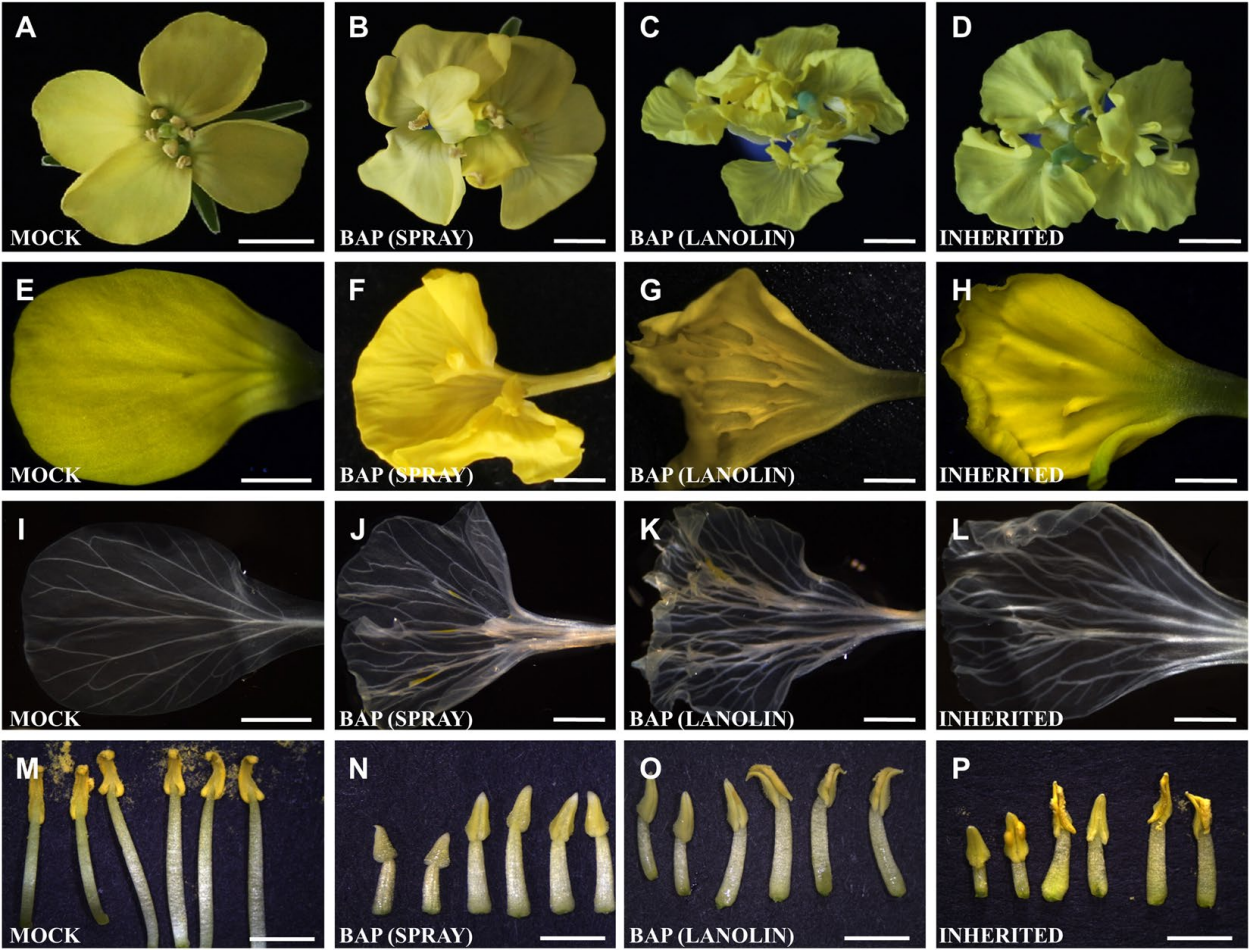
Phenotypes caused by exogenous BAP application in *Brassica napus* flower. (**A**,**E**,**I**,**M**) Mock-treated wild type flowers. (**B**,**F**,**J**,**N**) BAP-treated flowers by spraying. (**C**,**G**,**K**,**O**) BAP-treated flowers by lanoline. (**D**,**H**,**L**,**P**) Offspring of BAP-treated flowers. Scale bars: (**A**–**D**) 5 mm; (**E**–**L**) 2 mm; (**M**–**P**) 3.5 mm.

**Figure 3:**
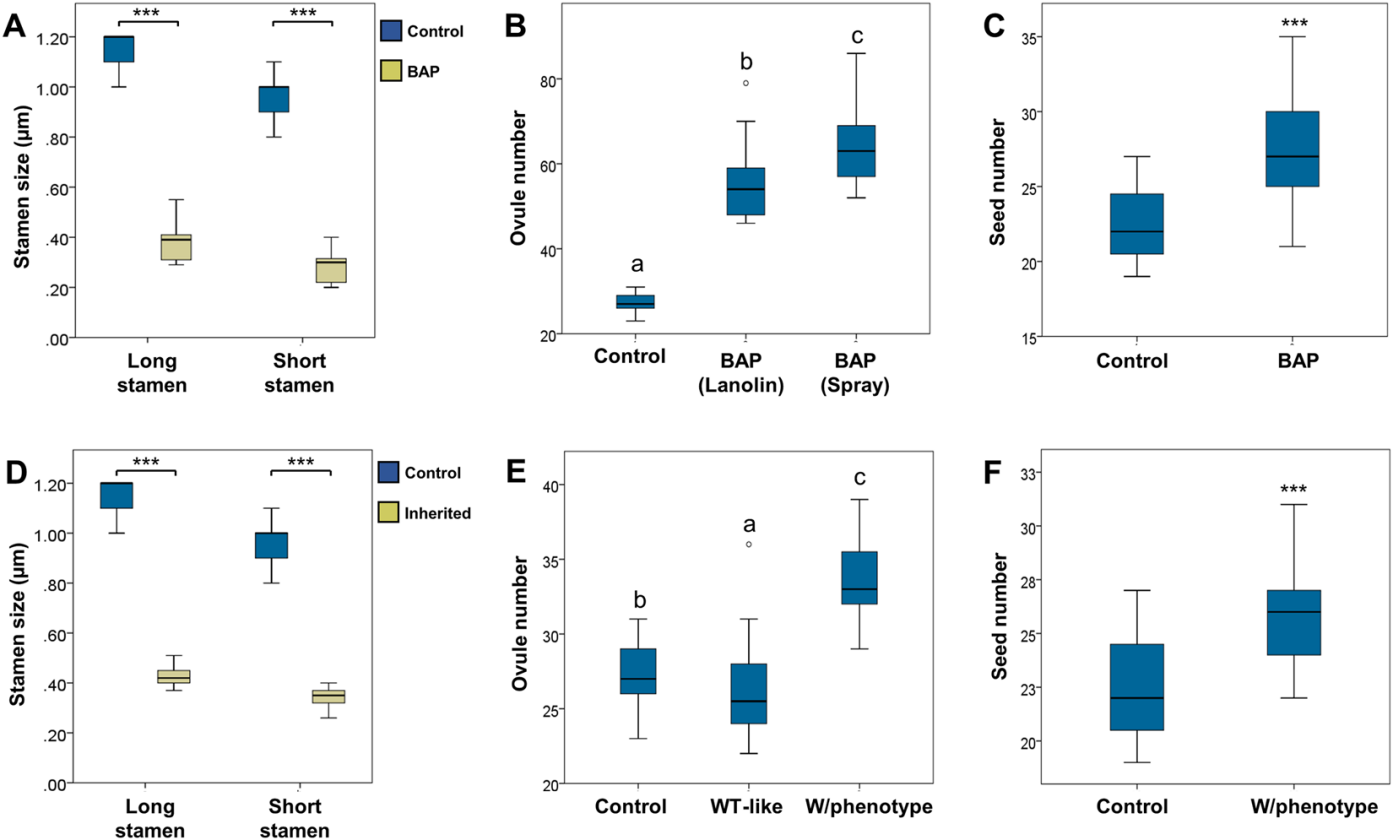
Effect of exogenous BAP application on stamen size and ovule and seed number in *Brassica napus*. (**A**) Stamen size in control and BAP-treated plants. (**B**) Number of ovules per gynoecium in control and BAP-treated plants. (**C**) Number of seed per fruit in control and BAP-treated plants. (**D**) Stamen size in control and the offspring of BAP-treated plants. (**E**) Number of ovules per gynoecium in control and the offspring of BAP-treated plants. (**F**) Number of seed per fruit in control and the offspring of BAP-treated plants. (**A**,**D**) ***P < 0.05 (t-student test). (**B**,**E**) Letters represent a statistical group, P < 0.001 (Kruskall-Wallis test). (**C**,**F**) ***P < 0.001 (Mann-whitney test). (**A**,**D**) *n* = 40 (control) and *n* = 80 (BAP and inherited); (**B**,**C**,**E**,**F**) *n* = 45.

During the phenotypic analysis we observed characteristics that were common between both treatments and others that were observed only in one of the treatments. In both treatments, the sepals had no visible morphological changes. However, several changes were observed in petals, which depended on the kind of treatment (Fig. 2A–C,E–G,I–K). Cytokinin-sprayed inflorescences showed flowers with increased petal size. This phenotype was not observed in lanolin-treated flowers. Furthermore, in both types of treatments, petals showed a conspicuous overgrowth of tissue that emerged from their adaxial side, with a more severe phenotype in lanolin-treated petals. Moreover, they had jagged margins (Fig. 2E–G). We cleared the petals with ethanol for more detailed observations and found that the petals in both treatments presented vasculature alterations. Whereas in untreated petals a main vascular bundle with organized secondary veins was seen, in the treated petals a less organized vasculature pattern was observed with more than one main vascular bundle (Fig. 2I–K). In both types of treatment, the stamens showed very short filaments. While control stamens were 1.15 (±0.07) and 0.97 (±0.08) mm long (long and short stamens, respectively), stamens of BAP-sprayed plants were only 0.37 (±0.06) and 0.28 (±0.05) mm long (long and short stamens, respectively), resulting in a statistical significant difference (Fig. 3A). Besides, anthers did not reach maturity uniformly. Only some parts of each anther were able to produce pollen (Fig. 2M–O). Anthers with mild phenotypes were able to produce pollen; however, that pollen was not able to reach the stigma due to the short length of the filaments, causing sterility.

In summary, these results indicate that *B. napus* has a cytokinin response similar to *A. thaliana* in sepals and stamens; while in petals it has extra phenotypes besides those reported for *A. thaliana*.

### Exogenous cytokinin application increases ovule number in *Brassica napus*

**Figure 4:**
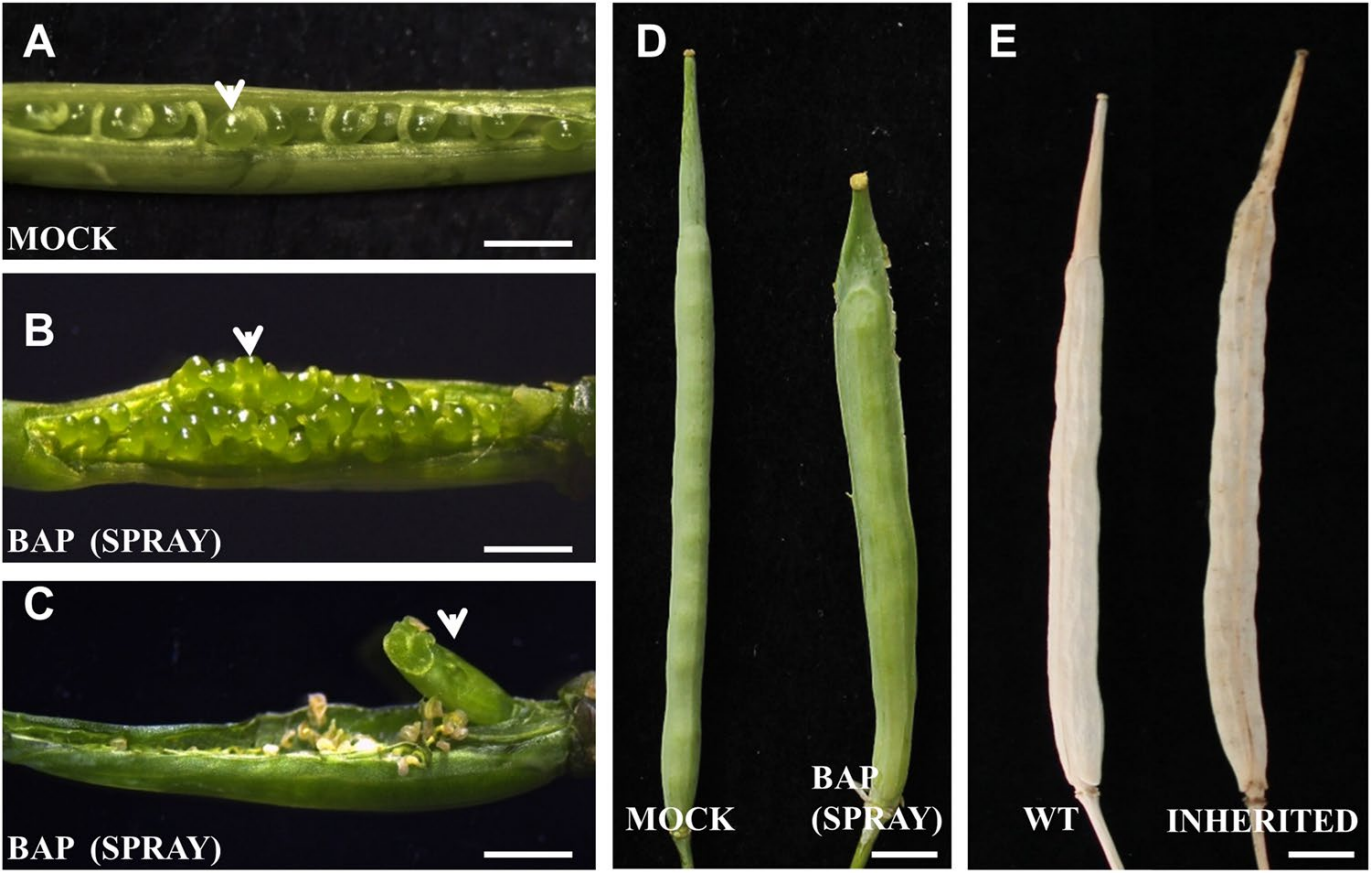
Phenotypes caused by exogenous BAP application in gynoecium and fruit of *Brassica napus*. (**A**) Mock-treated gynoecium where the ovules are shown (arrowhead). (**B**) BAP-treated gynoecium where the ovules are shown (arrowhead). (**C**) BAP-treated gynoecium where an extra carpeloid structure is shown (arrowhead). (**D**) Mock and BAP-treated green fruits that have reached their maximum size. (**E**) Mature fruit of a wild type plant and of an offspring plant that was BAP-treated showing an inherited phenotype. Scale bars: (**A**–**C**) 1 mm; (**D**,**E**) 5 mm.

An important feature to improve in canola as a crop, is its yield. Yield increase may be achieved in several ways and one of them is increasing the number of seeds produced per fruit. We tested whether the application of exogenous cytokinin was able to increase ovule number in *B. napus*. The number of ovules per gynoecium produced from inflorescences subjected to both types of treatment were analyzed and compared to the control. Untreated gynoecia produced 27.24 (±2.06) ovules, the cytokinin containing lanolin-treated gynoecia produced 54.95 (±7.34) ovules, and cytokinin sprayed gynoecia produced 64.64 (±9.39) ovules, resulting in a statistical significant difference (Figs 3B and 4A,B). These results clearly indicated that both treatments were able to at least double the number of ovules per gynoecium in *B. napus*, and that the spraying treatment had a slightly greater effect.

Next, we wanted to test whether this increase in ovule number could result in a higher number of seeds per fruit. Due to the cytokinin treatment, as described above, stamen development was affected resulting in semi-sterile anthers, therefore, we decided to hand-pollinate the treated gynoecia with pollen of untreated flowers.

The control plants (untreated) produced 22.60 (±1.55) seeds per fruit, while a treated and hand-pollinated gynoecium produced 26.82 (±3.95) seeds per fruit, which represents a statistical significant increase of 18% seed production (Figs 3C and 4D). The number of seeds per fruit was more variable between treated fruits than between control fruits. In control fruits we could find 20 to 25 seeds while in treated fruit found 21 to 35 seeds. This suggests that although a treated gynoecium is capable of producing more than 65 ovules, seed production is limited to a maximum of 35 seeds per fruit under our growth conditions. These results indicate that exogenous cytokinin is able to increase the number of seeds per fruit in *B. napus*, when pistils are pollinated manually.

### Exogenous cytokinin application causes ectopic outgrowth from the replum and increases replum size in *Brassica napus* and *Arabidopsis thaliana*

**Figure 5:**
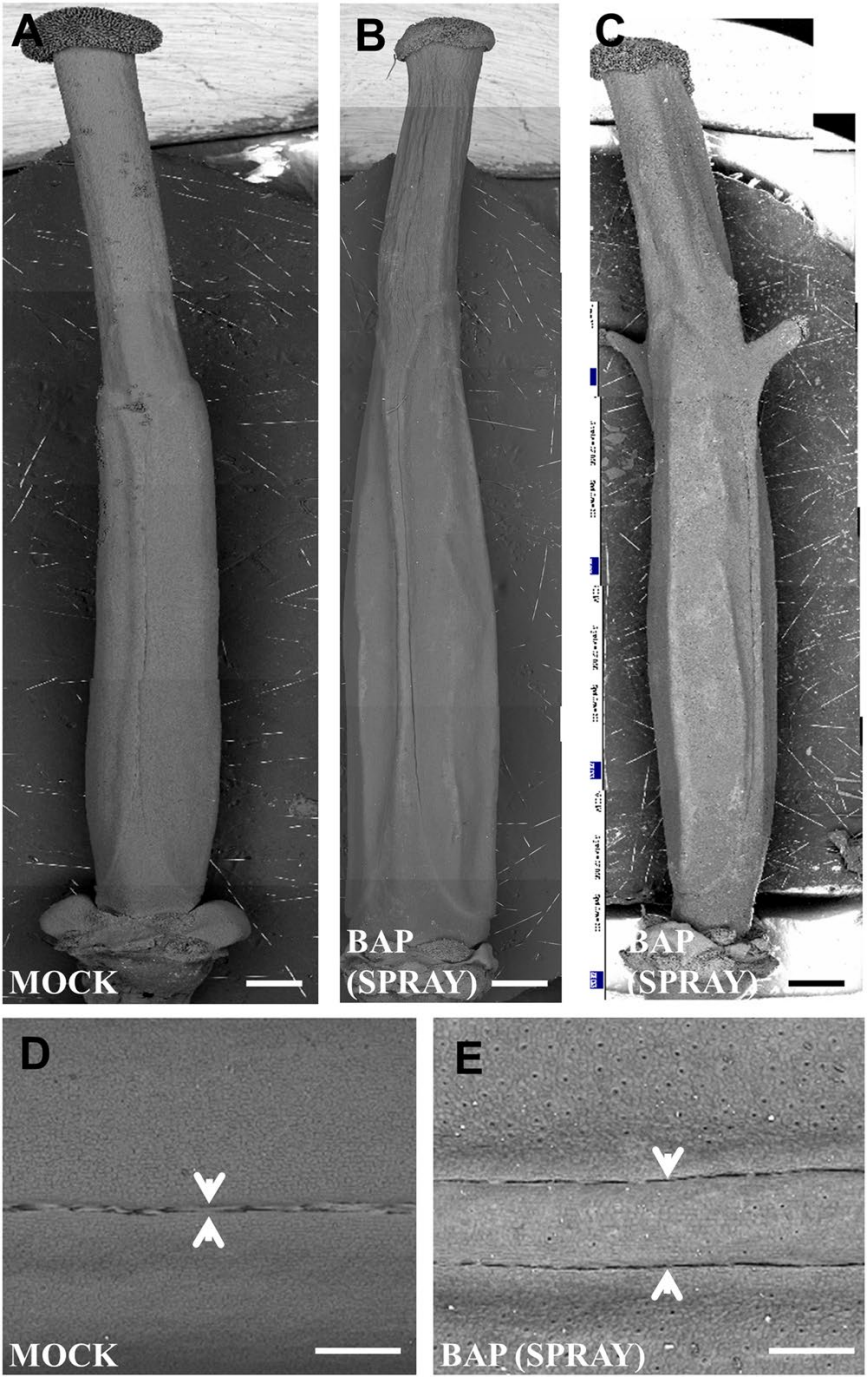
Exogenous BAP application affects replum development in gynoecium of *Brassica napus*. (**A**) Mock-treated gynoecium. (**B**,**C**) BAP-treated gynoecium that developed a wider replum. (**C**) BAP-treated gynoecium that developed extra tissues from the replum. (**D**) A zoom of a Mock-treated gynoecium where the replum is shown. (**E**) A zoom of a BAP-treated gynoecium where the replum is shown. Arrowheads indicate replum width. Scale bars: (**A**–**C**) 5 mm; (**D**,**E**) 200 µm.

As mentioned before, exogenous cytokinin application causes tissue proliferation that arises from the replum in *A. thaliana* gynoecia^22^. A similar phenomenon was observed in cytokinin-treated *B. napus* inflorescences. Ectopic proliferating tissue emerged from the repla with stigmatic tissue at the apical end; these phenotypes were observed with both types of treatment (Fig. 5A,C). Another phenotype observed, but only in the spray-treated gynoecia, was the development of a structure resembling a gynoecium that emerged from the base of the original gynoecium from inside where the gynoecium joins with the pedicel (Fig. 4C), this phenotype was not observed in *A. thaliana*.

**Figure 6:**
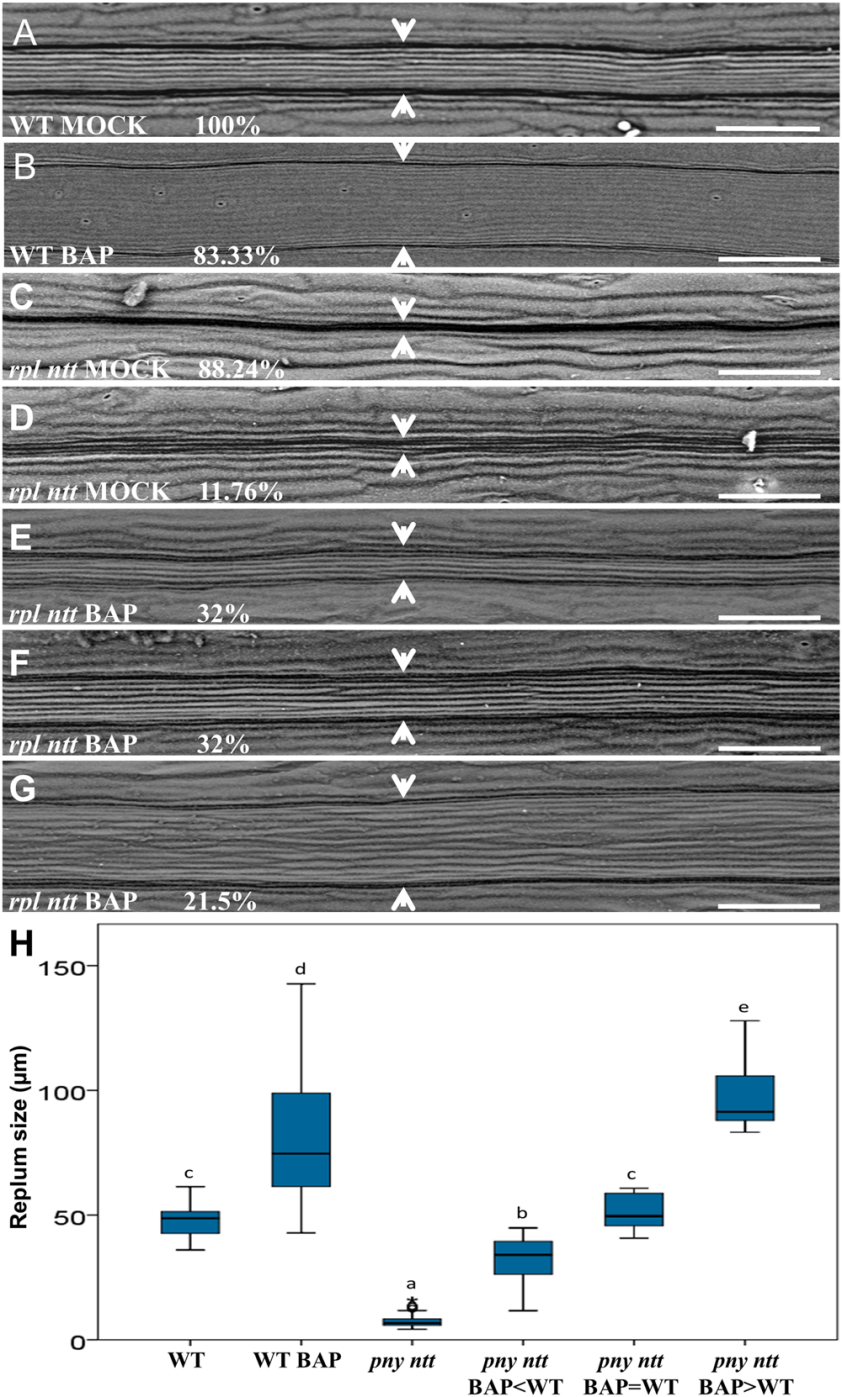
Exogenous cytokinin application increases replum size in *Arabidopsis thaliana*. (**A**) Mock-treated wild type gynoecium. (**B**) BAP-treated wild type gynoecium that developed a wider replum. (**C**,**D**) Mock-treated *rpl ntt* gynoecium that lacks or has a very narrow replum, respectively. (**E**–**G**) BAP-treated *rpl ntt* gynoecia where replum development is recovered, at different levels (arbitrary divided in three groups): The replum developed less than observed in WT (**E**); The replum developed as observed in WT (**F**); The replum developed more than observed in WT (**G**). (**H**) Replum size comparison of wild type (WT) and *rpl ntt* fruits of mock and BAP-treated plants (pny = *rpl*). Letters represent a statistical group, an ANOVA multiple range test was performed. Arrowheads indicate replum width. Scale bars: (**A**–**G**) 100 µm. (**A**,**B**) *n* = 24; (**C**,**D**) *n* = 17; (**E**–**G**) *n* = 28.

The replum development in *B. napus* is different with regard to *A. thaliana*, whereas in *A. thaliana* the replum has a certain width (Fig. 6A,H)^22^, in *B. napus* the replum is very narrow and is just visible as a thin line (Fig. 5A,D). Interestingly, when cytokinin was applied to *B. napus*, now a replum can be observed very similar as seen in *A. thaliana* (the same in both treatments) (Figs 5B,E and 6A). Actually, it has been reported that this very narrow replum can be explained by a point mutation in a *cis*-regulatory element of the transcription factor *REPLUMLESS* (*RPL*)^27^. In *A. thaliana*, a loss-of-function allele in *RPL* combined with a loss-of-function in the transcription factor *NO TRANSMITTING TRACT* (*NTT*) has severely altered replum development and also just a furrow line is visible (Fig. 6C,D,H)^28^. So, we wondered whether we could rescue replum development in the *A. thaliana rpl ntt* double mutant. We treated the *rpl ntt* double mutant inflorescences with a 100 µM BAP solution twice in the same day, three weeks after, the first three fruits produced by each treated inflorescence were analyzed. Strikingly, 85.5% of the *rpl ntt* fruits developed a replum, meaning that cytokinin rescued the phenotype (Fig. 6). From those, 32% of the treated *rpl ntt* fruits developed repla (32.58 ± 8.20) wider than the *rpl ntt* control (7.75 ± 3.16) but smaller than wild type (47.76 ± 5.76), 32% developed repla (51.21 ± 6.60) as wide as wild type, and 21.5% developed repla (98.17 ± 14.35) even wider than wild type fruits (47.76 ± 5.76) and BAP-treated wild type fruits (80.41 ± 24.88) (Fig. 6). These results indicate that cytokinin is necessary for replum development in both *B. napus* and *A. thaliana*.

### Cytokinin-induced phenotypic changes are inherited to the offspring in *Brassica napus*

In order to check whether the seed produced by the cytokinin-treated plants were viable, we sowed the seeds from fruits generated by hand-pollination of gynoecia with wild type pollen. The seeds germinated normally in soil and during the vegetative phase these plants showed a wild type phenotype. However, and to our surprise, during the reproductive phase, the flowers showed similar phenotypes as their parents (cytokinin-treated plants), although the offspring was not subjected to cytokinin treatment.

We decided to determine to what extent the observed phenotypes in the offspring plants were similar to the phenotypes of the cytokinin-treated plants.

The phenotypes of offspring flowers were presented in a gradient along the inflorescences, where the first five to eight flowers were indistinguishable from those treated flowers (Fig. 2A–D) while the following flowers increasingly resembled a wild type flower.

The offspring flowers showed an increase in the surface area of petals, also they had jagged edges and alterations in the vasculature as observed in cytokinin-treated flowers (Fig. 2E–L). Also, the stamens were affected, they presented shorter filaments than those of wild type flowers, anther maturation was not uniform and less pollen was produced, as seen in the cytokinin-treated parent plants (Figs 2M–P and 3D). The altered petal phenotypes as described before were also visible, which diminished in each new flower that was formed. Only the anthers maintained alterations till the end of the plant life cycle.

We also analyzed ovule number in the gynoecia of the offspring. Different phenotypes were observed (untreated-like and treated-like). The gynoecia resembling untreated wild type gynoecia produced 26.15 (±3.02) ovules, a similar number to the 27.24 (±2.06) of wild type gynoecia. However, the offspring gynoecia resembling cytokinin-treated gynoecia produced 33.52 (±2.77) ovules, a statistical significant increase of 23% compared to untreated wild type gynoecia (Fig. 3E). In order to determine whether this increase in ovule number resulted in an increase in the number of seeds, the offspring gynoecia were hand-pollinated and the resulting fruits produced 25.79 (±2.19) seeds per fruit, which represents a statistical significant increase of 14% seed production compared to untreated wild type fruits (Fig. 3F).

On the other hand, the wide replum phenotype and the overgrowth of tissue that emerged from repla were not observed in the gynoecia of offspring plants.

## Discussion

We studied the effect of cytokinin applications during flower and fruit development in *B. napus*. In general, exogenous cytokinin applications in *B. napus* flowers caused similar alterations to those observed in *A. thaliana* flowers^22^. However, it also caused alterations that have not been observed in *A. thaliana*. A striking difference was observed in petals. Contrary to that observed in *A. thaliana*, where the petals became slightly larger^22^, petals of treated flowers in *B. napus* drastically increased their size and we observed tissue proliferation on the adaxial side of the petal lamina and sometimes in the petal stalk. These results suggest that petals of *B. napus* are more sensitive to exogenous cytokinin application compared to petals of *A. thaliana*, and further suggest that cytokinin is capable of inducing proliferation and altering the growth axis in the petals of *B. napus*.

On the other hand, exogenous cytokinin application in *B. napus* caused an increase of more than twice the number of ovules per gynoecium. However, the number of seeds per fruit was increased only by 18%. This result indicates that in order to achieve a fruit with more seeds it is not enough to make a gynoecium with more ovules, suggesting that *B. napus* has a mechanism which limits the number of seeds that can be produced per fruit. It would be interesting to analyze whether this mechanism is determined by a genetic program or influenced by growth conditions such as nutrient availability.

During the last decades, scientists have been trying to increase crop yield by increasing endogenous cytokinin levels. Two different approaches have been followed: 1) increasing cytokinin biosynthesis by the ectopic expression of *ISOPENTENYL TRANSFERASE* (*IPT*) genes that catalyze the rate-limiting step in cytokinin synthesis or 2) reducing cytokinin degradation through down-regulation of *CYTOKININ OXIDASE/DEHYDROGENASE* (*CKX*) genes. For the first case, several promoters have been used, however, although some positive effects have been achieved, such transgenic plants also had both morphological and physiological negative effects^14–18^. Recently, it has been reported that transgenic canola (*B. napus*) plants expressing the *IPT* gene under the control of a modified version of the *AtMYB3*2 promoter showed increased yield^29^. The modified promoter lost activity in the roots^29,30^. The transgenic plants did not show any fitness penalty. Moreover, the plants showed a delay in leaf senescence, which may contribute to the increase in yield through the maintenance of photosynthetically active leaves for a longer period. Furthermore, the fruits showed reduced shattering, preventing seed loss in the field, which normally is an important problem with canola^29^. For the second approach, natural variations have been identified where some *CKX* genes are down-regulated or an RNAi strategy to silence these genes has been used^7,19,20^.

Based on our results and from literature, the use cytokinins to improve crop yield appears to be an attractive strategy. However, it is necessary to finely regulate cytokinin levels, since too elevated levels are detrimental for the plant. A good strategy to get more seeds per fruit would be to use a specific regulatory region to up-regulate *IPT* genes or to down-regulate *CKX* genes exclusively in the placental tissue of the gynoecium, in order to stimulate the meristematic activity of this tissue^21,25,31^. Moreover, this strategy could be combined with genetics or the use of specific regulatory regions that provide expression in the inflorescence meristem to increase the cytokinin concentration, resulting in the production of more fruits per plant^18,21,29^.

The ovary in *B. napus* and *A. thaliana* consists of two carpels, which are divided by a septum. The center of the septum differentiates into the transmitting tract and the abaxial part of the septum is called the replum^1,32^. In wild type *A. thaliana* plants, the silique has a replum that is around 8 cells wide. Certain mutants develop a more narrow replum such as the *rpl* mutant and the *rpl ntt* double mutant where most of their fruits have narrow or lack repla, respectively^28,33^. Interestingly, wild type *B. napus* fruits develop a narrow replum, resembling the *rpl ntt* double mutant^28^. This narrow replum has also been observed in fruits of *B. rapa* and linked to a single-nucleotide polymorphism between *A. thaliana*, *B. rapa*, and rice^27^. The polymorphism is in a conserved *cis*-regulatory element of the *RPL* gene, the same element that was identified to be important for reduced seed shattering in domesticated rice^34^. This single-nucleotide polymorphism, which decreases *RPL* expression, has been associated with the lack of a replum in the fruit^27^. Interestingly, we observed that the replum width of *B. napus* fruit could be recovered by cytokinin application. In *A. thaliana*, we also observed that replum development in fruits of the *rpl ntt* double mutant was rescued upon exogenous cytokinin application. These results indicate that cytokinin is an important component of replum development in both species. Additional support for this idea comes from experiments in *A. thaliana*, where we either increased or decreased cytokinin levels in the siliques, and observed increased or decreased replum width, respectively^22^. Moreover, because in *A. thaliana* replum recovery occurs in the absence of *RPL* and *NTT*, it suggests that cytokinin acts downstream of these genes. It would be interesting in future research to study the possible interactions between *RPL*, *NTT*, and cytokinin.

Plants are sessile organisms; thus, they are not able to escape adverse environmental conditions. However, in response to changing conditions they have a high phenotypic plasticity. This plasticity is due to changes in expression of many genes. In turn, the regulation of gene expression can occur at different levels, one is at the epigenetic level. Epigenetic modifications regulate gene expression without altering the DNA sequence, and this can be heritable^35^. Such modifications include DNA methylation, chromatin remodeling, histone modifications and non-coding RNAs^35^.

In this study, we observed that some of the obtained phenotypes due to exogenous cytokinin application in *B. napus* were inherited to the offspring. Because it is unlikely that cytokinin caused DNA mutations and the observed phenotypes were inherited transiently, they were most likely caused by epigenetic modifications.

One possible explanation is that somehow cytokinin caused changes in DNA methylation levels. Interestingly, the offspring of independently treated plants had the same phenotypes, meaning that the modifications were not random and that some genes are more prone to be altered upon cytokinin treatments.

Another interesting phenomenon was that not all the effects observed in the originally treated plants were inherited to the offspring. Altered petals, and increased number of ovules and seed were observed in the next untreated generation. However, the replum phenotype and ectopic proliferating tissue from the repla were not. This may suggest that the epigenetic modifications of specific loci are transient, while others are stable and transmitted to the next generation.

There are some reports that support the idea of possible changes in DNA methylation levels caused by cytokinins^36–38^. Such changes in DNA methylation seem to be associated with the S-adenosyl-L-homocysteine hydrolase (SAHH) enzyme, which is a key enzyme for maintenance of homeostasis methylation in eukaryotes. This enzyme catalyzes the conversion of S-adenosyl-L-homocysteine (SAH) into adenosine and L-homocysteine, thereby avoiding the SAH inhibitory effect in the methylation of DNA^39^. While the relationship between cytokinins and the SAHH is not totally understood so far, it has been reported that exogenous cytokinin application increases *SAHH1* expression and of at least three DNA methyltransferases in *A. thaliana*^38^. On the other hand, down-regulation of *SAHH1* causes DNA hypomethylation and increased amount of cytokinins in *A. thaliana* and Nicotiana^36–38,40^. In another study that links cytokinin and epigenetic modifications, Li and collaborators showed that a demethylated paternal genome or a reduction of H3K27me3 marks affected the expression level of *CYTOKININ OXIDASE 2* (*CKX2*), a gene that encodes an enzyme involved in cytokinin catabolism, in *A. thaliana* seeds^41^. This suggests a complex relationship between cytokinin and DNA methylation. Furthermore, it is not known whether cytokinins are able to increase or decrease DNA methylation levels, therefore more studies are required to elucidate this complex network.

Recently, it has been reported that in *A. thaliana* leaves the *BRAHMA* gene, which is part of SWI/SNF chromatin remodeling complex, interacts with the transcription factors *CIN-TCP* genes to jointly modulate cytokinin responses and hence promote determinate leaf growth^42^. *BRAHMA* also has been linked to floral organ development^43,44^. Interestingly, one of the phenotypes that were inherited to the offspring was related to an increase in petal size, which is a leaf-like structure, so it is possible that at least some of the inherited phenotypes in *B. napus* may be due to chromatin remodeling. Future studies should shed more light on these findings.

## Materials and Methods

### Plant Growth Conditions

In this study, *Brassica napus* and *Arabidopsis thaliana* ecotype Columbia plants were used. Plants were germinated in soil under long-day conditions (16–8 h, light–dark) in a growth chamber at 22 °C. One week after germination for *A. thaliana* and two weeks after germination for *B. napus*, the plants were transferred to the greenhouse with a temperature range from 22 to 28 °C, long-day conditions (13–11 h, light–dark approximately) and natural light. The *rpl-2 ntt* double mutant has been previously described^28^.

### Hormone Treatments

Treatments to *B. napus* plants were started one week after bolting, employing two different methods. In the first, the inflorescences were sprayed five days a week for three weeks, with a solution containing 200 µM BAP (6-Benzyl aminopurine; Duchefa Biochemie) and 0.02% Silwet L-77 (Lehle Seeds, Round Rock, TX, USA) or a mock solution containing only 0.02% Silwet L-77. In the second treatment, inflorescences were treated with a single application of lanolin containing 500 µM BAP or a mock treatment with lanolin alone. All treated plants with their respective controls were grown simultaneously under the same conditions. The flowers were analyzed after anthesis.

Hormone treatments to *A. thaliana* plants were performed one week after bolting. The inflorescences were dipped twice the same day in an eight-hour interval with a solution containing 100 µM BAP and 0.01% Silwet L-77 or a mock solution containing only 0.01% Silwet L-77. Three weeks after the treatment, the first three fruits produced by the main or secondary inflorescences were analyzed.

### Microscopy

Plant material was dissected and observed using a Leica EZ4 D stereomicroscope (Leica, Wetzlar, Germany). For scanning electron microscopy, plant tissue was collected and directly observed using a Zeiss EVO40 environmental scanning electron microscope (Carl Zeiss, Oberkochen, Germany) with a 20 kV beam, and the signal was collected using the BSD detector.

### Data availability

All data generated or analyzed during this study are included in this published article. All material used in this study is freely available.
